# Functional Investigation on Automotive Interior Materials Based on Variable Knitted Structural Parameters

**DOI:** 10.3390/polym12112455

**Published:** 2020-10-23

**Authors:** Mao Siyao, Su Liu, Zhang Peihua, Long Hairu

**Affiliations:** 1 College of Textiles, Donghua University, 2999 North Renmin Road, Songjiang District, Shanghai 201620, China; maosiyaodhu@163.com (M.S.); phzh@dhu.edu.cn (Z.P.); hrlong@dhu.edu.cn (L.H.); 2 Engineer Research Center of Technical Textile, Ministry of Education, Shanghai 201620, China

**Keywords:** weft-knitted structures, automotive interior, polymers, mechanical properties, fuzzy comprehensive evaluation

## Abstract

With the rapid development of technical textiles, more and more researchers have focused on developing high performance textiles to meet various needs. The automotive industry is a major market for technical textiles. Compared to other types of fabric, weft-knitted fabric has good extensibility and elasticity, as well as a hand-feel, and it is gradually becoming the preferred type of interior fabric for automobiles. This paper aims to develop an automotive fabric with good comfort and durability. Sixteen types of weft-knitted fabrics with eight different structures and two different materials (draw textured polyester and textured polyamide yarn) were fabricated using a computerized flat knitting machine. Their durability and level of comfort were examined by measuring the tensile and tear strengths, abrasion resistance and air permeability. A fuzzy comprehensive evaluation method was employed to compare the comprehensive properties of the fabric. The results indicated that the overall performance of DTPA fabric was better than DTPE fabric, and an optimum structure was selected for an automotive interior. Meanwhile, we found that the air permeability of the fabric could be increased by using tuck stitches and that the strength and dimensional stability of fabric could be increased by adding tuck stitches and weft-insert yarns. The findings contribute to the field of technical textiles and provide ideas for the development of high-performance textiles.

## 1. Introduction

Technical textiles have been regarded as textile manufactured for their technical and functional features, rather than for their decorative and aesthetic characteristics. The rapid development of technical textiles has made them be widely used in various fields, such as garments [1,2], the military [3], the medical field [4], and transportation, and it has aroused the interest of a wide variety of researchers [5,6,7]. One of the largest markets for technical textiles is the automobile industry, which uses an average of 20 kg for each of the 45 million or so cars produced globally each year [8]. With advancements in living standards, cars have gradually become an indispensable part of life worldwide. Meanwhile, advancements in textile technologies mean that fabrics have begun to replace leather as the preferred automotive interior material, and using textiles is also considered to be environmentally friendly, functional and economical. Compared to woven and warp-knitted fabrics, weft-knitted fabrics can, to a large extent, provide a two-way stretch and have good elongation and elasticity [9]. Hence, automakers are increasingly favoring the usage of weft-knitted fabrics for different interior components, such as seat covers, dashboard covers, side panels, etc. Some researchers have extended their studies to focus on improving the performance of fabrics used in the automotive industry, such as resistance to abrasion [10,11,12], tensile and tear properties [9,13,14], thermal stability [15,16], flame retardance [17,18] and physiological properties [19]. Researchers have also focused on other functional properties of automotive interior materials, such as sound absorption [20,21,22,23] and water and oil repellency [24]. However, most of them focus on a certain performance, and there are few studies on the comprehensive properties of knitted fabrics. Meanwhile, details of the fabric structure have not been provided in related articles, and the relationships between fabric structure and the properties of textiles used for the automotive interior are still not clear.

Hence, this study proposes the use of weft-knitted fabrics with different structures that are fabricated on a computerized flat knitting machine for automotive upholstery and investigates their mechanical properties and level of comfort. According to previous research, some important properties of these fabrics are examined, such as their tensile strength, abrasion resistance, tear strength and air permeability, which are usually the essential factors examined for automotive upholstery.

Polyester and polyamide are currently the most used types of materials in the automotive industry [25]. The properties of polyester, which make it an ideal material for automotive interiors, include high tensile and tear strengths, low moisture absorbency, and good abrasion and UV resistance. Meanwhile, the prominent advantage of using polyamide is its excellent abrasion resistance; therefore, two types of materials were selected to knit the samples. The comprehensive influence of different structures and the type of yarn on the fabric was also analyzed. This study will contribute to the development of knitted fabric that is used for automotive interiors.

## 2. Materials and Methods

### 2.1. Materials

**Figure 1:**
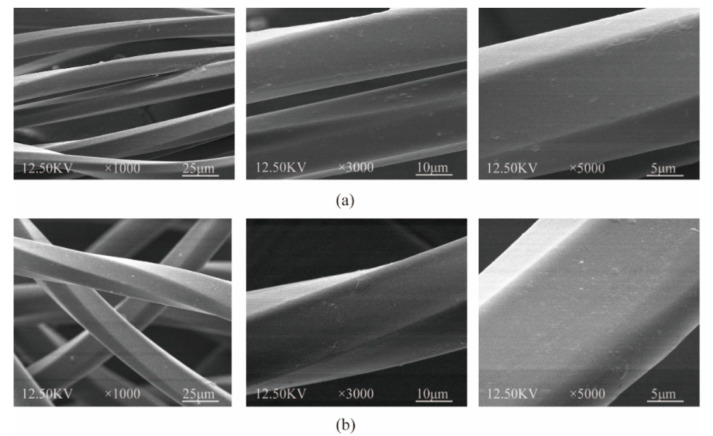
E-SEM micrographs of (**a**) DTPE and (**b**) DTPA magnified 1000, 3000 and 5000 times.

Polyester and polyamide are currently the most used types of materials in the automotive industry. Furthermore, compared to common yarn, draw texturing yarn (DTY) has a better resilience, hand feel and glossiness. Therefore, 300D draw textured polyester (DTPE) and draw textured polyamide yarn (DTPA) are used in this study to fabricate the automotive interior fabric samples, as shown in Table 1, and the SEM micrographs of DTPE and DTPA using a QUANTA environmental scanning electron microscope (FEI, Check, Hillsboro, OR, USA) are shown in Figure 1a,b.

### 2.2. Samples Preparation

Eight types of knit structures (four types of single knit fabrics and four types of double knit fabrics) were used as the samples. The single knit structures are plain knit, 1 × 1 plain knit, plain knit with weft-inlay and double pique, numbered by 1, 2, 3 and 4. The double knit structures are interlock, variable half milano, interlock with weft-inlay and variable half cardigan, numbered by 5, 6, 7 and 8. All of the samples are produced on a STOLL ADF 32W (Reutlingen, Germany) computerized flat knitting machine 7.2 gg (with the corresponding knitting parameters of eight structures listed in Table 2).

**Figure 2:**
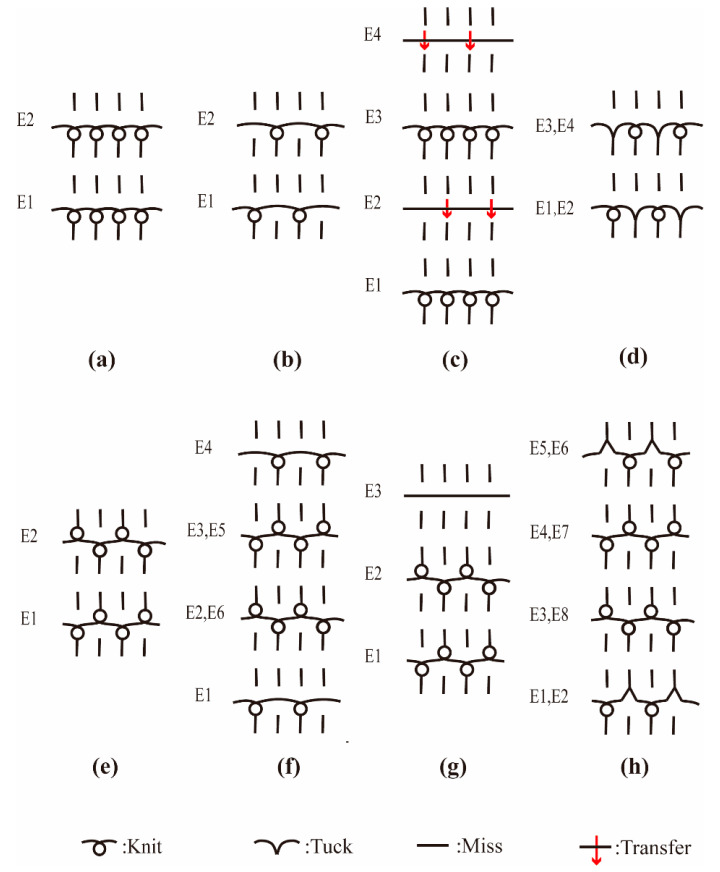
Loop diagrams of different structures: (**a**) plain knit; (**b**) 1 × 1 plain knit; (**c**) plain knit with weft-inlay; (**d**) double pique; (**e**) interlock; (**f**) variable half milano; (**g**) interlock with weft-inlay; and (**h**) variable half cardigan.

**Figure 3:**
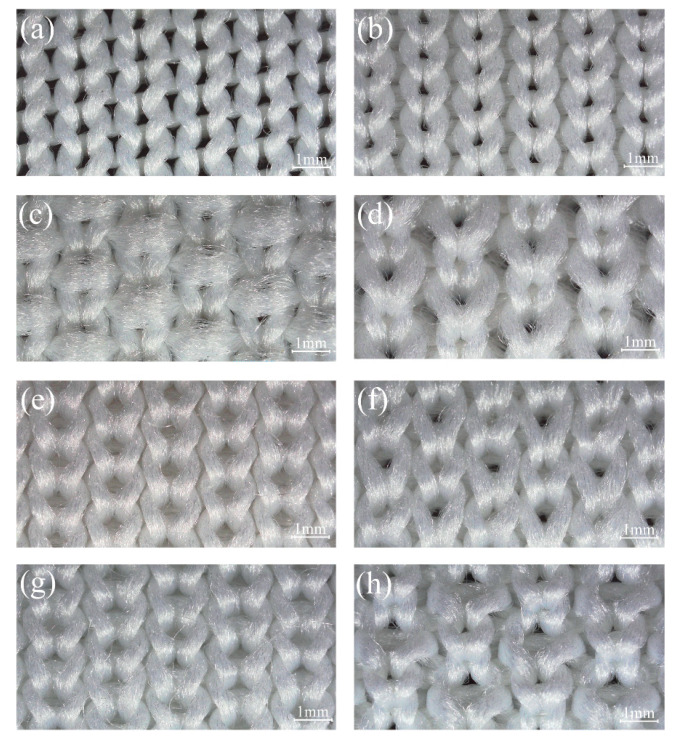
Fabric photographs captured by microscope: (**a**) plain knit; (**b**) 1 × 1 plain knit; (**c**) plain knit with weft-inlay; (**d**) double pique; (**e**) interlock; (**f**) variable half milano; (**g**) interlock with weft-inlay; and (**h**) variable half cardigan.

**Figure 4:**
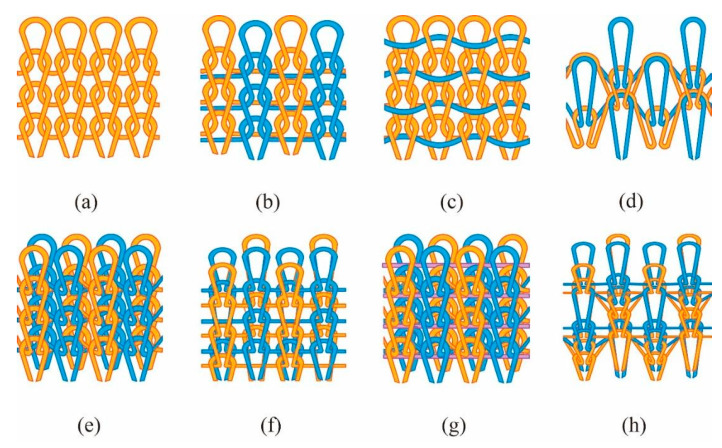
Simulation images of different structures: (**a**) plain knit; (**b**) 1 × 1 plain knit; (**c**) plain knit with weft-inlay; (**d**) double pique; (**e**) interlock; (**f**) variable half milano; (**g**) interlock with weft-inlay; and (**h**) variable half cardigan.

The characterizations of the fabric samples are listed in Table 3, and the loop diagrams are shown in Figure 2a–h. Images of eight structures captured by a microscope (ECLIPES LV 100N POL, Tokyo, Japan) and the simulation images are shown in Figure 3a–h and Figure 4a–h. To obtain the optimal hand feel and fabric quality, two yarn ends were selected to knit the DTPE fabrics, while three yarn ends were used to knit DTPA fabrics, considering the individual properties of the two polymer materials.

### 2.3. Testing Methods

**Figure 5:**
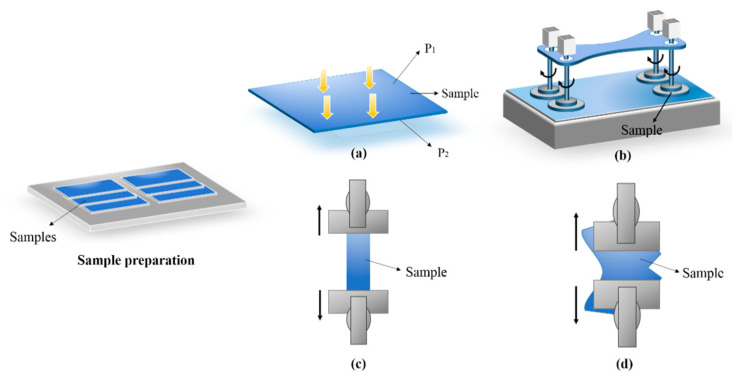
The experimental illustration: (**a**) air permeability testing; (**b**) abrasion resistance testing; (**c**) strip testing; (**d**) tear strength testing.

Before the testing was carried out, all of the samples were stored in standard atmospheric conditions of 20 ± 2 °C and 65 ± 2% relative humidity (RH) for 24 h in accordance with standard ISO 139:2005. The experimental illustration is shown in Figure 5a–d.

#### 2.3.1. Air Permeability Testing

The air permeability testing was carried out on a YG461E air permeability tester (WenZhou Fangyuan Instrument Co., Ltd., Wenzhou, China) under standard atmospheric conditions. The samples were cut into dimensions of 20 cm × 20 cm in accordance with standard ISO 9237:1995. The pressure difference on each sample was 200 Pa. Each sample was tested ten times in different positions, and the average was calculated and used.

#### 2.3.2. Abrasion Resistance Testing

**Figure 6:**
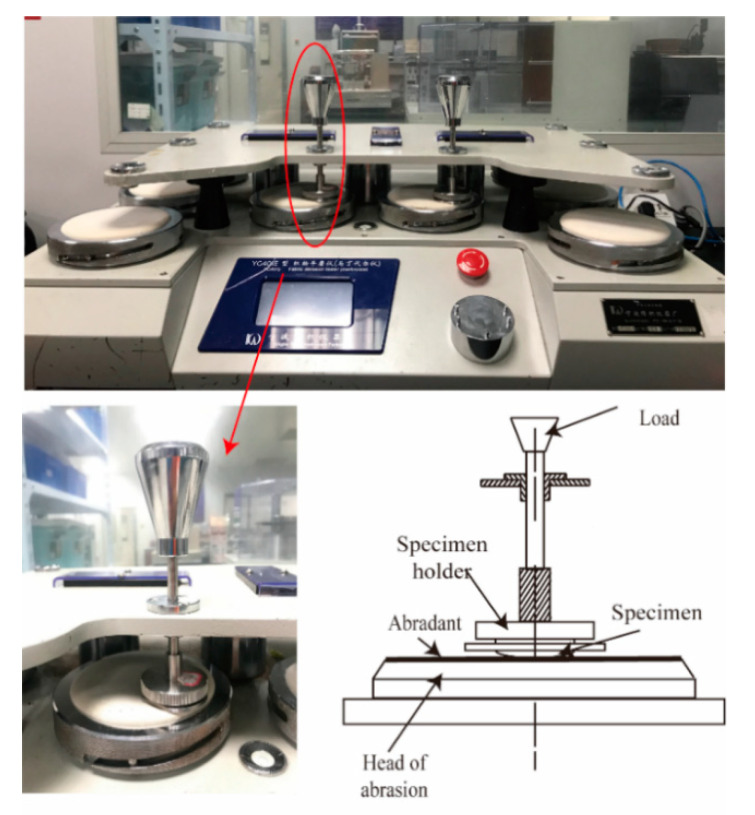
Rubbing head of the Martindale tester.

The abrasion resistance testing was carried out on a YG041E fabric abrasion tester (Ningbo Textile Instrument Factory, Ningbo, China). Figure 6 shows the illustration for the rubbing head of the abrasion and pilling tester. Three circular specimens with a diameter of 38 mm were extracted from each sample in accordance with standard GB/T 21196.3-2007 (China). The weight loss of each sample after 20,000 cycles was calculated.

#### 2.3.3. Strip Testing

To determine the tensile strength, strip testing was carried on the YG028 Electronic fabric strength tester (Ningbo Dahe Instrument Co., Ltd., Ningbo, China) in accordance with standard GB/T 3923.1-2013 (China). For every structure, five samples were prepared at a uniform size of 50 mm × 200 mm (width × length) in the course and wale directions, respectively. The distance between the clamps was set at 100 mm, and the tester was operated at a loading rate of 100 mm/min. The average was calculated and used.

#### 2.3.4. Tear Strength Testing

**Figure 7:**
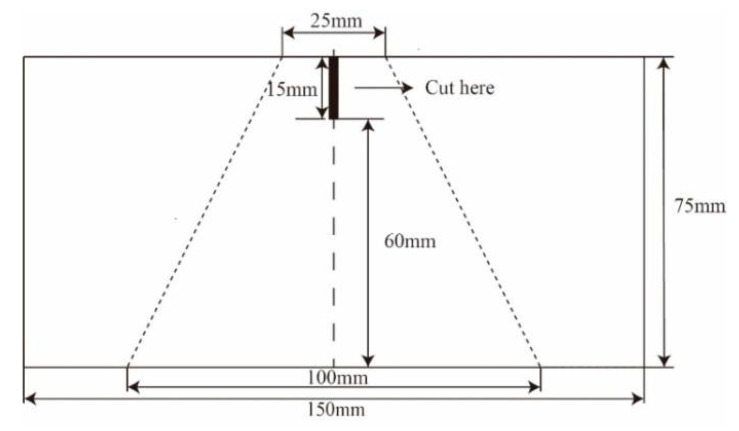
Sample specifications for the tearing test.

The tear strength testing was also carried out on the YG028 Electronic fabric strength tester (Ningbo Dahe Instrument Co., Ltd., Ningbo, China). Five trapezoid-shaped pieces were cut from the samples in both the course and wale directions in accordance with standard GB/T 3917.3-2009 (China), and the specifics of the samples are shown in Figure 7. A tear with a length of 15 mm was made in advance. The distance between the clamps was set at 25 mm, and the loading rate was set at 100 mm/min. Each sample was tested five times, and the average was calculated and used.

## 3. Results and Discussion

### 3.1. Air Permeability

**Figure 8:**
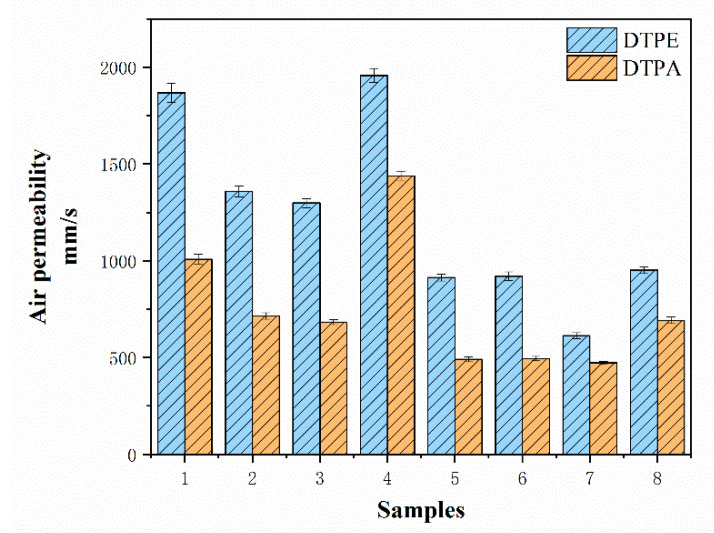
Air permeability results of different structures.

The results for the air permeability of the knit fabric samples are presented in Figure 8 and Table 4. It can be observed that the air permeability of DTPE samples is 29.7–90.0% higher than of DTPA samples. This is because the polyamide in the DTPA samples contributes to the higher elasticity of the samples so that the fabric has a higher stiffness, which reduces the porosity. At the same time, it can be observed that there are significant differences in the air permeability due to different structures. Specifically, A4 and B4, and A8 and B8 have the highest air permeability among single jersey samples and double jersey fabrics respectively. It can be up to 1.5–2.0 times higher than for other structures. The reason for this is because the used tuck stitches can form holes on the surface of the fabric, which allows more air to pass through the surface of the fabric. On the other hand, the air permeability of A3 and B3, and A7 and B7, which have a weft inlaid structure, decreases by 12.1–28.8% in comparison to other structures, as the weft-inlaid yarn will not form loops in fabrics and air permeability is therefore inhibited. In addition, the single jersey samples show a higher air permeability than the double jersey fabrics, which are thicker and therefore do not readily allow air to pass through.

**Figure 9:**
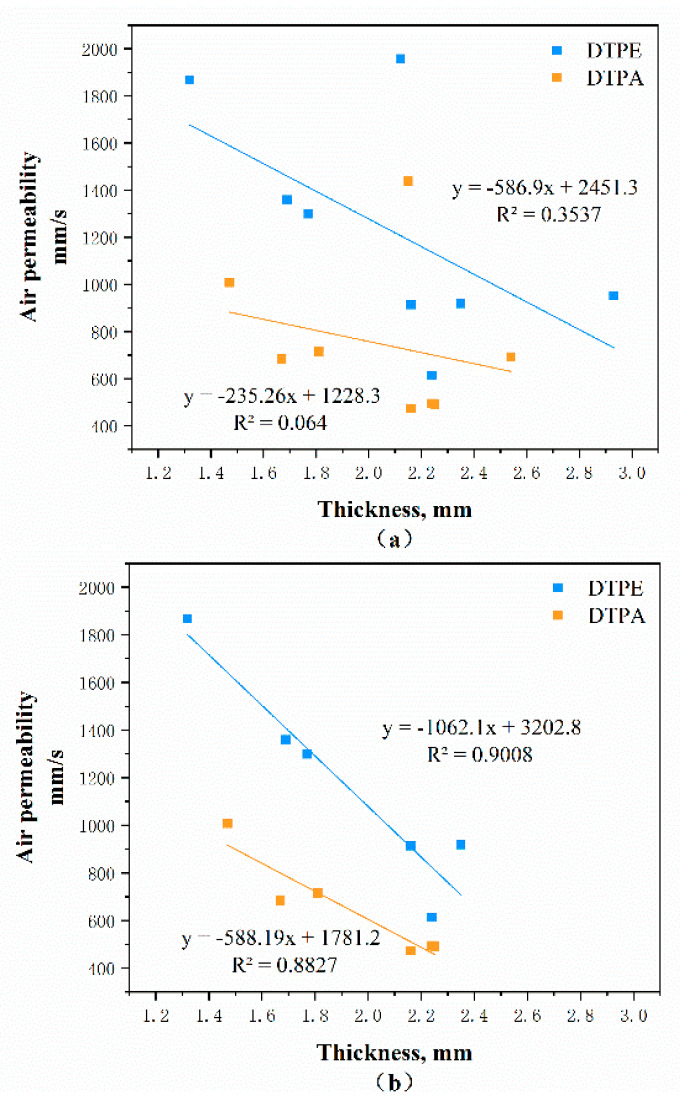
Plotted relationship between air permeability and thickness: (**a**) all samples, and (**b**) when eliminating tuck stitches (A4 and B4, and A8 and B8).

Therefore, aside from the material and structure, the thickness of fabric can also influence air permeability; see Figure 9. There is no obvious linear relationship in Figure 9a between the air permeability and thickness due to the particularities of the tuck stitches. When eliminating the tuck stitches (A4 and B4, and A8 and B8), the thickness of the fabric influences the amount of air flow that passes through the fabric, as depicted in Figure 9b, which shows an inverse relationship (r = −0.88 and r = −0.90, respectively). Thus, it can be concluded that the air permeability of the fabric is decreased with an increase in the thickness of the fabric.

### 3.2. Abrasion Resistance

**Figure 10:**
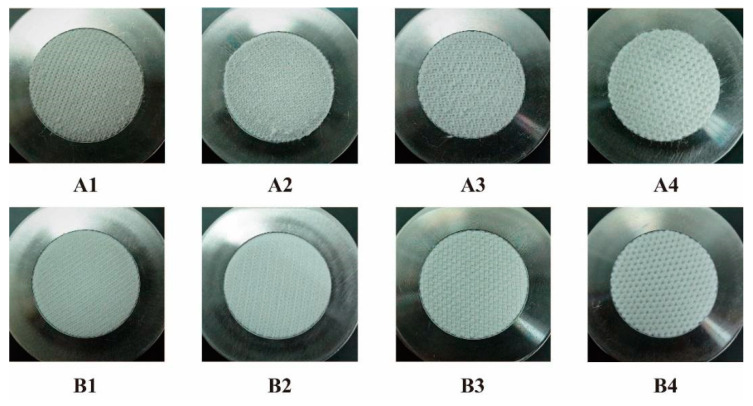
Single jersey fabrics after 20,000 cycles of testing: **A1**–**A4** for DTPE and **B1**–**B4** for DTPA.

**Figure 11:**
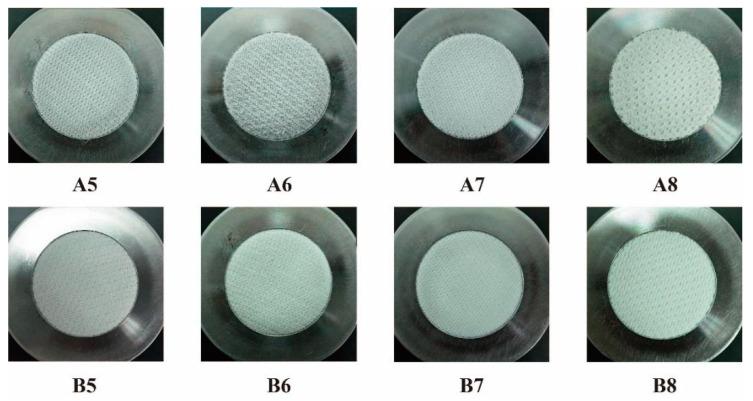
Double jersey fabrics after 20,000 cycles of testing: **A5**–**A8** for DTPE and **B5**–**B8** for DTPA.

A test of the abrasion resistance of the samples showed that DTPE and DTPA offered a good resistance against abrasion resistance, as no holes formed on the surface after 20,000 cycles of testing. Figure 10 and Figure 11 show the surface of the single jersey and double jersey samples after the abrasion test, respectively.

The figures show that the DTPA samples have a better resistance to abrasion than the DTPE samples do. After 20,000 cycles of testing, the surfaces of the DTPE samples show obvious pilling, and the partial area is not very intact, especially for the sample with the weft that is inlaid (A3). On the other hand, while the surfaces of the DTPA samples show some discoloration, their structure is still largely intact and only a few hairs appear on the surface. The reason for this result is that polyamide (DTPA) has an excellent abrasion resistance. Meanwhile, compared to the double jersey fabrics, the single jersey fabrics have a poor abrasion resistance, and there are obvious signs of wear on their surface.

**Figure 12:**
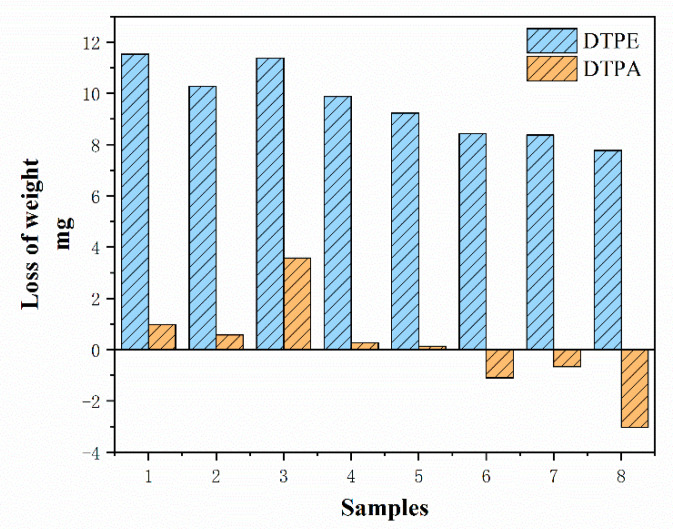
Mass loss of samples after 20,000 cycles of testing.

Figure 12 and Table 4 show the mass loss of the samples. The mass loss of the DTPE samples is 3.2–71.0 times more than that of the DTPA samples. Among the eight different types of fabric structure, A3 and B3 have higher mass losses (11.37 mg and 3.57 mg) due to the presence of floating yarns that can be easily pulled out. Furthermore, the mass loss of the single jersey samples is higher than that of the double jersey samples, as the latter are thicker and have a higher mass per unit area. It can also be observed that the weight change of B6 to B8 is negative. The reason for this unexpected finding might be that the abrasion resistance of the samples is higher than the abradant itself and that the particles of the abradant rubbed off onto the samples and increased their weight.

**Figure 13:**
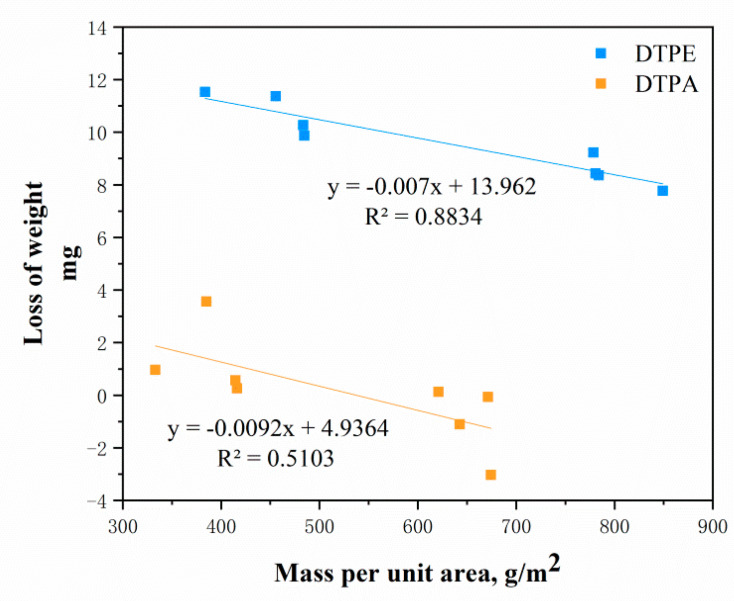
Relationship between mass loss and mass per unit area.

A correlation analysis was carried out (Figure 13), showing that the abrasion resistance of the samples had an obvious linear relationship with the mass per unit area (r = 0.51 and r = 0.88, respectively). It can be concluded that the abrasion resistance of the samples increases with an increase in the mass per unit area of the fabric.

### 3.3. Breaking Strength

**Figure 14:**
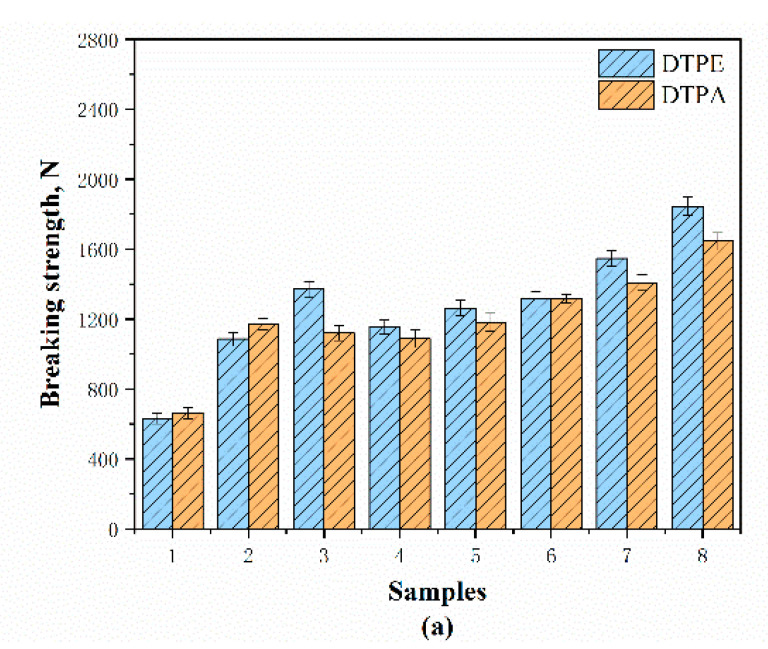

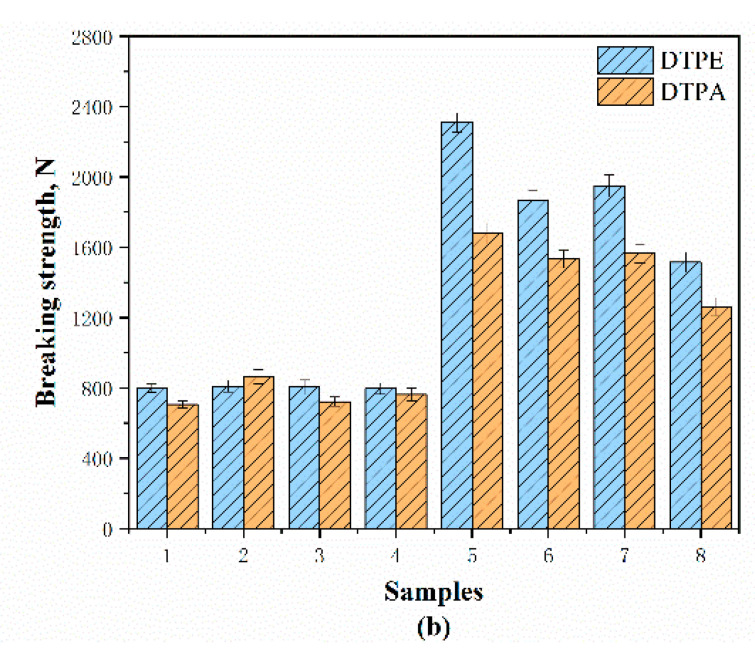
Breaking strength of samples along different directions: (**a**) course and (**b**) wale directions.

**Figure 15:**
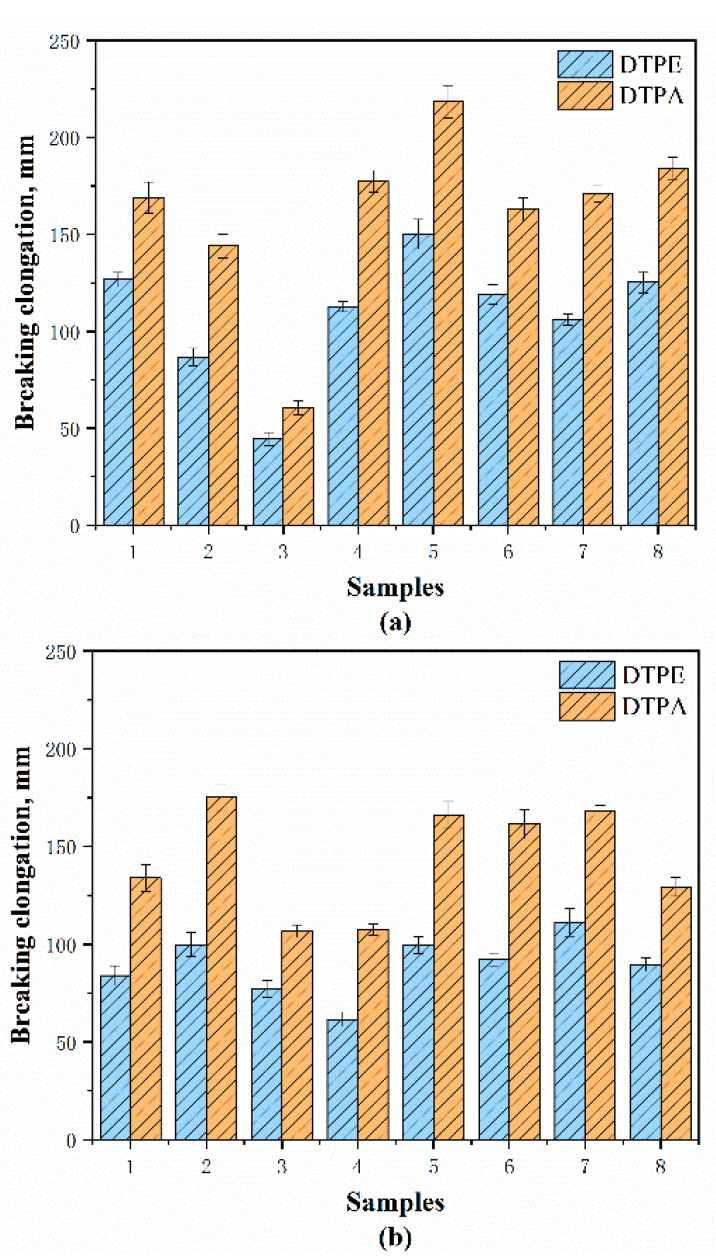
Breaking elongation of samples along different directions: (**a**) course and (**b**) wale directions.

According to standard GB/T 33276-2016 (China), the breaking strength of automotive interior fabric should be higher than 350 N, and after the test, it can be seen that all the samples are up to the standard. Figure 14a,b and Figure 15a,b show the breaking strength and breaking elongation of the samples in different directions, and the difference between the different structures can be observed. Meanwhile, the breaking strength and elongation of the same structure in different directions are also different.

The figures show that the DTPE samples have a higher breaking strength than the DTPA samples because the polyester yarns in the DTPE samples are higher in strength. At the same time, the good elasticity of the DTPA samples makes the breaking elongation be 1.3–2.2 times higher than for DTPE samples. Furthermore, the double jersey samples have a higher breaking strength than the single jersey samples because knitted yarns on both sides allow a tight connection.

Specifically, A1 and B1 have the lowest breaking strengths in the course (631.02 N and 660.90 N) and wale directions (798.48 N and 705.80 N), in which the loops can easily transfer the stress when the samples are subjected to external force. They also have a high breaking elongation in both directions. Meanwhile, A2 and B2 have a higher strength and dimension stability than A1 and B1, due to the presence of float yarns behind the stitches. Therefore, the samples with a weft-inlaid structure (A3 and B3, and A7 and B7) have a higher strength and lower breaking elongation in the course direction. It can be observed that A5 and B5 have the highest breaking strength in the wale direction (2309.54 N and 1680.60 N) because the yarns crisscross back and forth, and two rib stitches bind each other, which increases the difficulty of fabric detachment. However, A5 and B5 also have a higher breaking elongation in the weft direction than the other samples, as there are float yarns and tuck stitches added.

**Figure 16:**
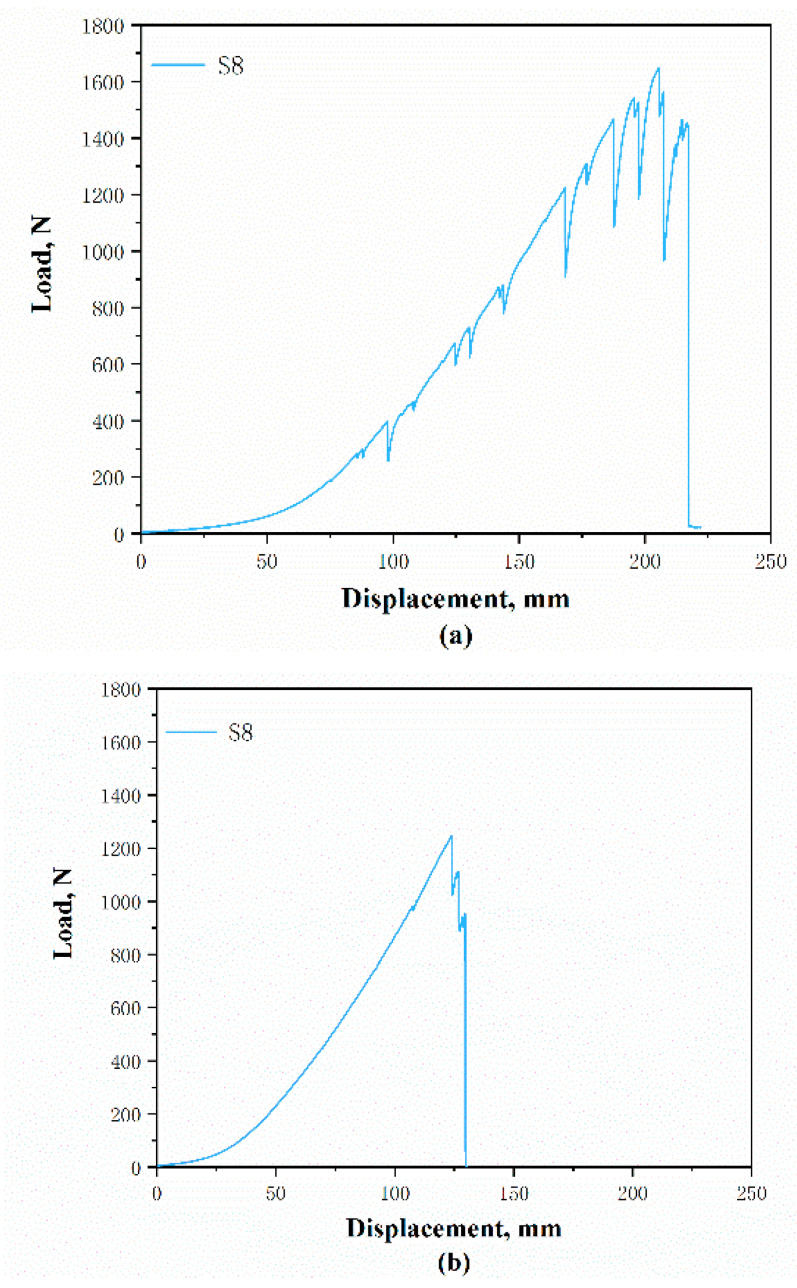
Plotted tensile displacement-load of DTPA S8 along: (**a**) course and (**b**) wale directions.

The tensile displacement-load curves of different structures are similar, so for the convenience of comparison, only one of their curves is selected. Figure 16a,b shows the tensile displacement-load curves of the DTPA S8 fluctuate in different directions. In the wale direction, the strength of the samples gradually increases linearly and then suddenly declines. This is because in the initial stages of the testing, the stitches began to deform and the loops in the wale direction were forced at the same time. Then, the yarns began to stretch until they finally ruptured. It was observed in the experiment that the tensile fracturing of the fabric in the wale direction took place almost immediately, but the stitches were still robust apart from being elongated.

**Figure 17:**
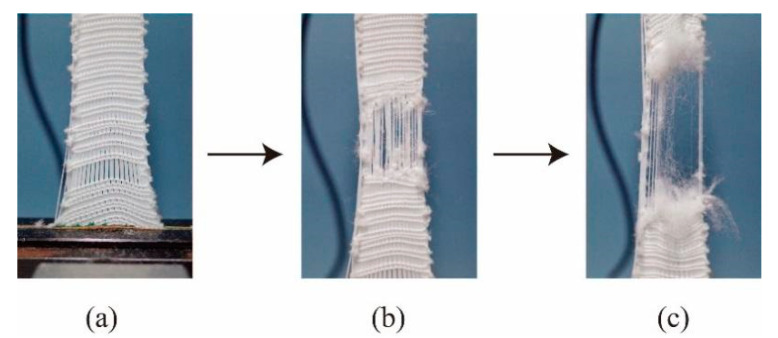
Breaking process of fabric in the course direction: (**a**) stitch slipping, (**b**) some rupturing and (**c**) ruptured.

The trend of the curve in the course direction shows fluctuating increases compared to that in the wale direction. This is because when the samples are subjected to stress, the stitches begin to fray, as shown in Figure 17a, which leads to a reduction in strength. Some parallel yarns can be found which begin to stretch, thus increasing the strength of the sample again. Therefore, there are fluctuations and many small peaks plotted. With an increased elongation, the yarns in the course direction rupture one by one until the fabric is completely damaged which can be seen in Figure 17b,c.

### 3.4. Tearing Strength

**Figure 18:**
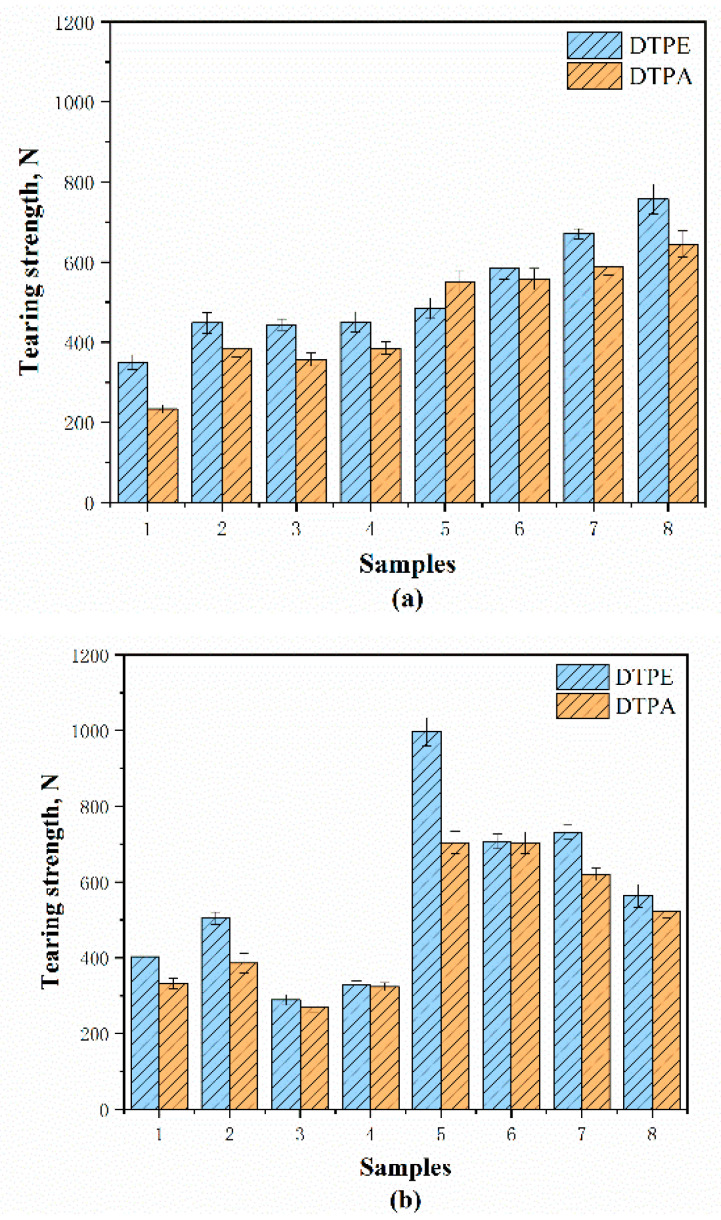
Tearing strength of samples along: (**a**) course and (**b**) wale directions.

Figure 18 and Table 4 show the tearing strength of the samples in the different directions. According to standard GB/T 33276-2016 (China), the tearing strength of automotive interior fabric should be higher than 30 N. It can be seen in Figure 18a,b and Table 4 that all of the samples meet the requirement at more than 9.0–33.2 times.

Figure 18 shows that the tearing strength of the DTPE samples is 10–50% higher than that of the DTPA samples, as the DTPE yarn has a higher strength. Specifically, the tearing strength of A5 and B5 in the wale direction (996.64 N and 704.27 N) is the highest as they have the highest stitch density, which prevents the yarns from slipping. On the other hand, A8 and B8 show the lowest tearing strength (563.55 N and 523.54 N) among the double jersey samples in the wale direction. This is because, in the wale direction, the tuck stitches can easily allow tearing as opposed to the loop stitches and the tearing strength is decreased by 15.7–43.5% in comparison to other double jersey structures. In addition, the single jersey fabrics show a lower resistance to tearing than the double jersey fabrics, which have more interlacing between the yarns.

**Figure 19:**
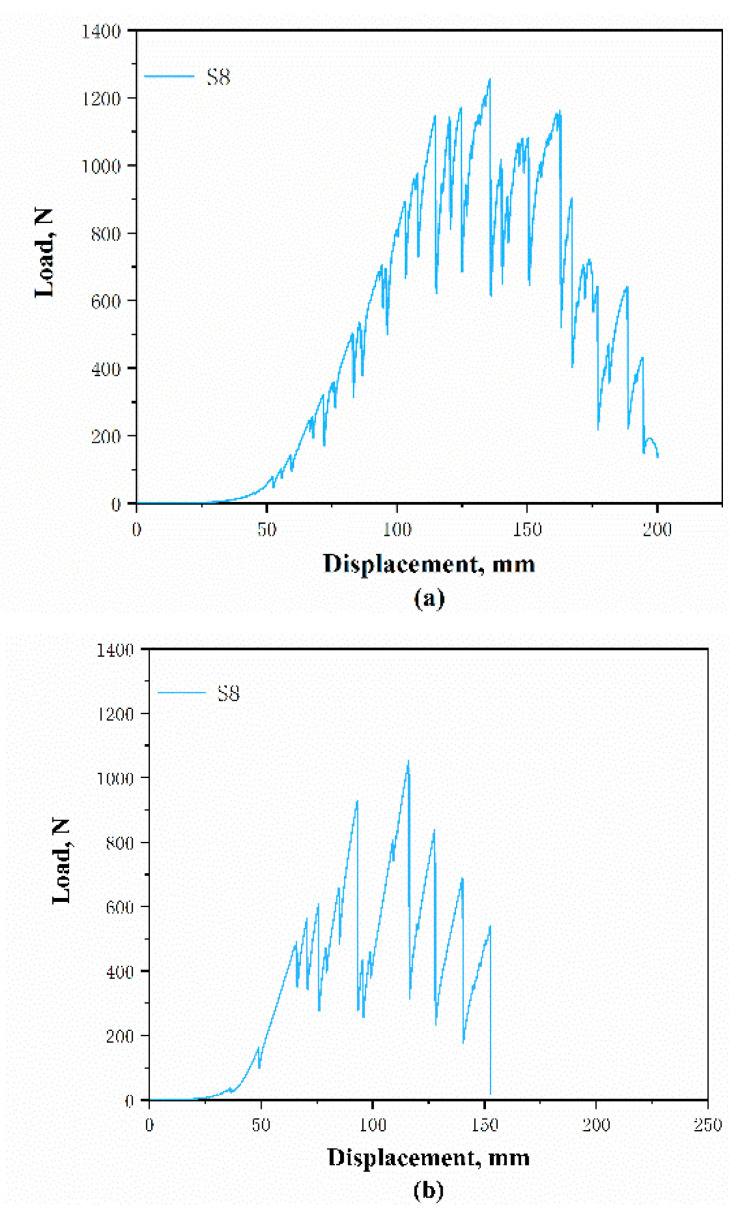
Plotted tear displacement load of DTPA S8 along: (**a**) course and (**b**) wale directions.

Similar to the stretch curves, the tear displacement-load curves of the DTPA S8 in the course and wale directions are selected and shown in Figure 19a,b. It can be observed that the tear displacement-load curves differ with the direction. The curves in the wale direction have fewer peaks compared to those in the course direction. The load between the peaks and valleys is also higher. This is because, when tearing the samples along the wale direction, the sample is torn from a prepared perforation and the stitches near the tear are subjected to force. Each time a decline in strength is shown on the curve, it can very likely be attributed to the breaking of the yarns. On the other hand, slipping of the course yarns will take place, which can also cause a strength decline in the process with the tearing of the samples along the course direction. Then, the stitch yarns gradually break until the samples are thoroughly damaged.

### 3.5. Comprehensive Evaluation of Utility Performance of Knitted Fabric

The fuzzy comprehensive evaluation aims to make an overall evaluation of the objects affected by various factors [26]. The general steps of the fuzzy comprehensive evaluation method are as follows: The first step is to establish parameters that reflect the performance of the object and construct the weight vector in a suitable way. Then, the fuzzy comprehensive evaluation matrix is established. Finally, the evaluation matrix and weight are combined to obtain the comprehensive evaluation result.

Eight types of weft-knitted fabrics with different structures and two different materials were analyzed. The air permeability, mass loss, breaking strength and tearing strength were selected as the indices for the comprehensive evaluation, and their test results are shown in Table 4.

First, the factor gather was set as *U*, *U* = (*U1, U2, U3, U4, U5, U6*), and *U1* to *U6* represent air permeability, mass loss, breaking strength in the course direction, breaking strength in the wale direction, tearing strength in the course direction and tearing strength in the wale direction, respectively.

Then, the following equations were used to establish the comprehensive evaluation matrix *R* [27]:

*r_ij_* = (*X_ij_* − *X_imin_*)/(*X_imax_* − *X_imin_*) (1)

*r_ij_* = (*X_imax_* − *X_ij_*)/(*X_imax_* − *X_imin_*) (2)

where *r_ij_* is the element of the judgment matrix *R*, *r_ij_* ∈ [0,1]; *X_ij_* is the text value of the *i*th index for the *j*th sample; *X_imax_* is the maximum value of the *i*th index and *X_imin_* is the minimum value of the *i*th index. Among the test results, when the higher the value is, the better the performance is, Equation (1) is used; otherwise, Equation (2) is used. The judgement matrix *R* obtained from the above equation is as follows:

$$R^{T}=[\begin{array}{cccccc} 0.940 & 0.000 & 0.000 & 0.058 & 0.222 & 0.183\\ 0.597 & 0.087 & 0.374 & 0.065 & 0.410 & 0.323\\ 0.556 & 0.011 & 0.609 & 0.061 & 0.402 & 0.027\\ 1.000 & 0.114 & 0.432 & 0.056 & 0.415 & 0.083\\ 0.297 & 0.158 & 0.522 & 1.000 & 0.480 & 1.000\\ 0.301 & 0.213 & 0.563 & 0.723 & 0.673 & 0.602\\ 0.095 & 0.217 & 0.754 & 0.775 & 0.835 & 0.635\\ 0.323 & 0.258 & 1.000 & 0.504 & 1.000 & 0.404\\ 0.360 & 0.725 & 0.025 & 0.000 & 0.000 & 0.084\\ 0.163 & 0.753 & 0.445 & 0.100 & 0.285 & 0.160\\ 0.142 & 0.547 & 0.403 & 0.010 & 0.236 & 0.000\\ 0.650 & 0.773 & 0.377 & 0.036 & 0.290 & 0.074\\ 0.012 & 0.783 & 0.455 & 0.608 & 0.605 & 0.598\\ 0.015 & 0.867 & 0.566 & 0.516 & 0.619 & 0.597\\ 0.000 & 0.838 & 0.640 & 0.536 & 0.676 & 0.483\\ 0.148 & 1.000 & 0.835 & 0.348 & 0.786 & 0.349 \end{array}]$$

In this work, the method of the questionnaire is adopted to determine the weight coefficient of each index. The investigated people are experts in the field of knitted fabrics and related technicians. After the questionnaires were integrated, the weight vector *A* was shown as follows:

*A* = (0.2550,0.3670,0.1055,0.1055,0.0835,0.0835) (3)

Thus, the final comprehensive evaluation result is calculated as follows:

*Q = A·R =* (0.280,0.292,0.252,0.390,0.418,0.397,0.388,0.453,0.368,0.413,0.300,0.524,0.503,0.538,0.528,0.624) (4)

From the above results, it can be seen that the comprehensive evaluation of the 16 samples from high to low is B8, B6, B7, B4, B5, A8, A5, B2, A6, A4, A7, B1, B3, A2, A1 and A3. Through the questionnaire survey, it is found that abrasion resistance is a very important property in automotive fabric, especially in the fabric used for seat covers. Therefore, the comprehensive evaluation of DTPA fabric is significantly higher than that of DTPE.

In the same way, eight samples of DTPE and DTPA were evaluated respectively, and the results are as follows. For DTPE fabrics, the results of the comprehensive evaluation from high to low is A8, A6, A7, A5, A4, A2, A1 and A3. Meanwhile, for DTPA fabrics, the results of the comprehensive evaluation from high to low is B8, B6, B7, B4, B5, B2, B1 and B3. It can be seen from the results that the effects of different structures on the performance are nearly the same. Compared with single jersey fabric, double jersey fabric has a better comprehensive performance, and the best performance of the eight structures is S8 (variable half cardigan structure).

## 4. Conclusions

This study evaluated the mechanical properties and level of comfort offered by sixteen kinds of fabric samples with eight different structures and two different materials. Experiments were carried out to investigate their air permeability, abrasion resistance, and breaking and tearing strengths. The results are summarized as follows:

1. The air permeability of DTPE fabric is 29.7–90% higher than that of DTPA fabric. Among the eight different structures, the fabric with tuck stitches can increase the air permeability by 50–100%, while those structures with inlaid yarn can reduce the air permeability by 12.1–28.8%;
2. Compared with DTPE fabric, DTPA fabric has a better wear resistance. After 20,000 cycles of abrasion testing, there were only a few hairs on the surface of the DTPA samples, and the structure was still intact, while the DTPE samples showed obvious pilling. A comprehensive comparison of the wear and mass loss shows that S3 (plain knit with weft-inlay) has the worst abrasion resistance due to the presence of floating yarns on the surface;
3. The good elasticity of DTPA makes the breaking elongation be 1.3–2.2 times higher than for the DTPE samples. The double jersey structures have higher breaking and tearing strengths as the yarns are more tightly connected, especially the S5 (interlock). Meanwhile, the addition of weft-inlaid yarns and tuck stitches can enhance the dimensional stability and strength of the fabric;
4. The comprehensive evaluation shows that the comprehensive performance of DTPA fabrics is higher than that of DTPE, and among the eight different structures, S8 (variable half cardigan) is more suitable for automotive interior fabrics;

In summary, an appropriate structure and materials should be used to meet the mechanical requirements and level of comfort demanded of automotive interiors. This study has contributed to providing some guidance in the production of materials for automotive interior fabrics.

## Figures and Tables

**Table 1:** Specification of materials used in automotive interior fabrics.

Material	Yarn Count	Color	Provider
DTPE	300D/96F	White	PM Yarns and Textiles
DTPA	300D/96F	White	PM Yarns and Textiles

**Table 2:** Knitting parameters of different structures.

Structure No.	NP Value	Knitting Speed (mm/s)	Yarn Tension (N)	Pulling Value
S1	11.3	0.55	2.5	4.0
S2	11.3	0.55	2.5	4.0
S3	11.3	0.50	2.5	4.5
S4	11.3	0.55	2.5	4.5
S5	10.0	0.55	2.5	5.5
S6	10.0	0.55	2.5	5.5
S7	10.0	0.50	2.5	5.5
S8	10.0	0.55	2.5	5.5

**Table 3:** Characterizations of the fabric samples.

Sample No.	Material	Yarn Count	CPC ^1^	WPC ^1^	Thickness (mm)	Weight (g/m^2^)
A1	DTPE	300D × 3	7.6	9.8	1.32	383.64
A1			8.4	8.8	1.69	483.60
A3			6.4	10.0	1.77	455.56
A4			5.4	8.8	2.12	484.72
A5			7.2	9.2	2.16	778.44
A6			7.4	7.2	2.35	780.80
A7			6.0	10.0	2.24	784.40
A8			5.8	6.8	2.93	849.04
B1	DTPA	300D × 2	7.2	12.0	1.47	333.28
B2			8.0	9.6	1.81	414.72
B3			7.2	10.4	1.67	385.12
B4			6.0	9.2	2.15	416.40
B5			6.8	12.8	2.25	621.04
B6			7.2	8.4	2.24	642.56
B7			6.4	10.4	2.16	671.32
B8			5.6	7.2	2.54	674.44

^1^ CPC denotes courses per centimeter, and WPC denotes wales per centimeter.

**Table 4:** The test results of various properties for the samples.

Samples No.	Air Permeability (mm/s)	Mass Loss (mg)	Breaking Strength in Course Direction (N)	Breaking Strength in Wale Direction (N)	Tearing Strength in Course Direction (N)	Tearing Strength in Wale Direction (N)
A1	1867.53	11.53	631.02	798.48	349.94	402.84
A2	1358.68	10.27	1085.60	810.14	448.45	504.57
A3	1298.69	11.37	1370.32	803.75	443.91	289.21
A4	1957.20	9.87	1155.68	796.20	450.71	329.88
A5	913.47	9.23	1264.16	2309.54	485.02	996.64
A6	919.24	8.43	1314.97	1865.92	585.95	707.61
A7	613.04	8.37	1546.68	1949.c	670.91	731.55
A8	951.89	7.77	1845.08	1514.55	757.60	563.55
B1	1007.18	0.97	660.90	705.80	233.41	331.17
B2	715.23	0.57	1171.48	865.60	382.97	385.70
B3	683.54	3.57	1119.63	722.24	357.26	269.80
B4	1437.42	0.27	1088.23	763.58	385.55	323.75
B5	490.61	0.13	1182.93	1680.60	550.29	704.27
B6	495.20	−1.10	1318.13	1532.82	557.99	703.67
B7	472.59	−0.67	1407.82	1564.63	587.76	620.92
B8	692.45	−3.03	1645.19	1263.42	645.37	523.54
